# Development of an age-scalable 3D computational phantom in DICOM standard for late effects studies of childhood cancer survivors

**DOI:** 10.1088/2057-1976/ab97a3

**Published:** 2020-09-29

**Authors:** Aashish C Gupta, Suman Shrestha, Constance A Owens, Susan A Smith, Ying Qiao, Rita E Weathers, Peter A Balter, Stephen F Kry, Rebecca M Howell

**Affiliations:** 1 Department of Radiation Physics, The University of Texas MD Anderson Cancer Center, Houston, Texas, United States of America; 2 The University of Texas MD Anderson Cancer Center UTHealth Graduate School of Biomedical Sciences, Houston, Texas, United States of America

**Keywords:** computational phantoms, late effects, pediatric phantoms, dose reconstruction

## Abstract

**Purpose:**

We previously developed an age-scalable 3D computational phantom that has been widely used for retrospective whole-body dose reconstructions of conventional two-dimensional historic radiation therapy (RT) treatments in late effects studies of childhood cancer survivors. This phantom is modeled in the FORTRAN programming language and is not readily applicable for dose reconstructions for survivors treated with contemporary RT whose treatment plans were designed using computed tomography images and complex treatment fields. The goal of this work was to adapt the current FORTRAN model of our age-scalable computational phantom into Digital Imaging and Communications in Medicine (DICOM) standard so that it can be used with any treatment planning system (TPS) to reconstruct contemporary RT. Additionally, we report a detailed description of the phantom’s age-based scaling functions, information that was not previously published.

**Method:**

We developed a Python script that adapts our phantom model from FORTRAN to DICOM. To validate the conversion, we compared geometric parameters for the phantom modeled in FORTRAN and DICOM scaled to ages 1 month, 6 months, 1, 2, 3, 5, 8, 10, 15, and 18 years. Specifically, we calculated the percent differences between the corner points and volume of each body region and the normalized mean square distance (NMSD) between each of the organs. In addition, we also calculated the percent difference between the heights of our DICOM age-scaled phantom and the heights (50th percentile) reported by the World Health Organization (WHO) and Centers for Disease Control and Prevention (CDC) for male and female children of the same ages. Additionally, we calculated the difference between the organ masses for our DICOM phantom and the organ masses for two reference phantoms (from International Comission on Radiation Protection (ICRP) 89 and the University of Florida/National Cancer Institute reference hybrid voxel phantoms) for ages newborn, 1, 5, 10, 15 and adult. Lastly, we conducted a feasibility study using our DICOM phantom for organ dose calculations in a commercial TPS. Specifically, we simulated a 6 MV photon right-sided flank field RT plan for our DICOM phantom scaled to age 3.9 years; treatment field parameters and age were typical of a Wilms tumor RT treatment in the Childhood Cancer Survivor Study. For comparison, the same treatment was simulated using our in-house dose calculation system with our FORTRAN phantom. The percent differences (between FORTRAN and DICOM) in mean dose and percent of volume receiving dose ⩾5 Gy were calculated for two organs at risk, liver and pancreas.

**Results:**

The percent differences in corner points and the volumes of head, neck, and trunk body regions between our phantom modeled in FORTRAN and DICOM agreed within 3%. For all of the ages, the NMSDs were negliglible with a maximum NMSD of 7.80 × 10^−2^ mm for occiptital lobe of 1 month. The heights of our age-scaled phantom agreed with WHO/CDC data within 7% from infant to adult, and within 2% agreement for ages 5 years and older. We observed that organ masses in our phantom are less than the organ masses for other reference phantoms. Dose calculations done with our in-house calculation system (with FORTRAN phantom) and commercial TPS (with DICOM phantom) agreed within 7%.

**Conclusion:**

We successfully adapted our phantom model from the FORTRAN language to DICOM standard and validated its geometric consistency. We also demonstrated that our phantom model is representative of population height data for infant to adult, but that the organ masses are smaller than in other reference phantoms and need further refinement. Our age-scalable computational phantom modeled in DICOM standard can be scaled to any age at RT and used within a commercial TPS to retrospectively reconstruct doses from contemporary RT in childhood cancer survivors.

## 1. Introduction

Cancer survivors whose treatment included radiation therapy (RT) are at risk for developing RT-related late effects (⩾5 years after diagnosis) (Travis *et al* 2011). Survivors of childhood cancer are at particularly high risk because of high survival rates (>84%) and long-life expectancy (Armstrong *et al* 2016, Turcotte *et al* 2017, Gibson *et al* 2018, Howlader *et al* 2019). Retrospective epidemiologic studies of cancer survivor cohorts investigate the relationship between RT dose to specific organs or body regions and the risk of subsequent late effects (Travis *et al* 2011). Such studies typically use computational phantoms to retrospectively reconstruct doses throughout patients’ bodies (Lee *et al* 2010, Travis *et al* 2011, Xie and Zaidi 2014, Howell *et al* 2019) by recomputing the radiation field doses on a phantom. For cohorts that include survivors of childhood cancers, phantoms should be scalable to the age or size of the patient at the time of RT.

For over two decades (>120 studies), the MD Anderson Late Effects Group has used an age-scalable computational phantom to reconstruct dose to organs throughout the body for large cohorts of childhood cancer survivors treated with conventional two-dimensional (2D) historic RT (Howell *et al* 2019). This phantom is currently coded in the FORTRAN 95 programming language and can only be used with co-planar beam geometries, which were standard in 2D planning. Furthermore, its current format does not support instantaneous or three-dimensional (3D) visualization. These limitations were acceptable for previous studies with cohorts treated with historic 2D RT. However, cancer survivors now include individuals treated with contemporary RT, e.g., 3D conformal RT, intensity- and volumetric-modulated RT, and particle therapy. For these individuals, complex treatment plans were designed using patients’ computed tomography (CT) images within commercial treatment planning systems (TPS). Unlike in the 2D treatment planning era, dose to organs near the target volume are readily calculable as part of the treatment plans and therefore, will not need to be retrospectively recalculated. However, doses to distant organs will still need to be retrospectively reconstructed on computational phantoms, because CT images used for treatment planning may not be available for epidemiologic studies, and even if they are, the CT data will be limited to anatomy near the target volume and will not include distant structures that are typically of interest in such epidemiologic studies.

For retrospective whole-body dose reconstructions, the ‘missing’ anatomy could be supplemented by registering a patient’s planning CT(s) with a computational phantom scaled to the age at RT. The MD Anderson Late Effects Group computational phantom is well suited for this purpose because (1) it is the most widely used phantom for late effects studies of historic RT and using this same phantom for studies involving contemporary RT will facilitate direct comparison of results between historic and modern studies; and (2) it can be uniquely scaled to any arbitrary age or height whereas other computational phantoms are limited to specific selected ages. However, the MD Anderson Late Effects Group computational phantom is programmed in FORTRAN language, which is not compatible for registration with patients’ Digital Imaging and Communications in Medicine (DICOM) standard CT images in commercial TPSs. Therefore, the primary objective of this study was to adapt the current model of our age-scalable computational phantom from the FORTAN language to DICOM standard for use within any commercial TPS, thereby facilitating epidemiologic studies of contemporary radio-therapy. Additionally, we report a detailed description of our age-based scaling functions, information that was not reported in our previous publications. Note that hereafter, we use phantom, FORTRAN, and DICOM for computational phantom, FORTRAN language, and DICOM standard, respectively.

## 2. Methods

### 2.1. Phantom modeled in FORTRAN language (baseline phantom description)

**Figure 1.**
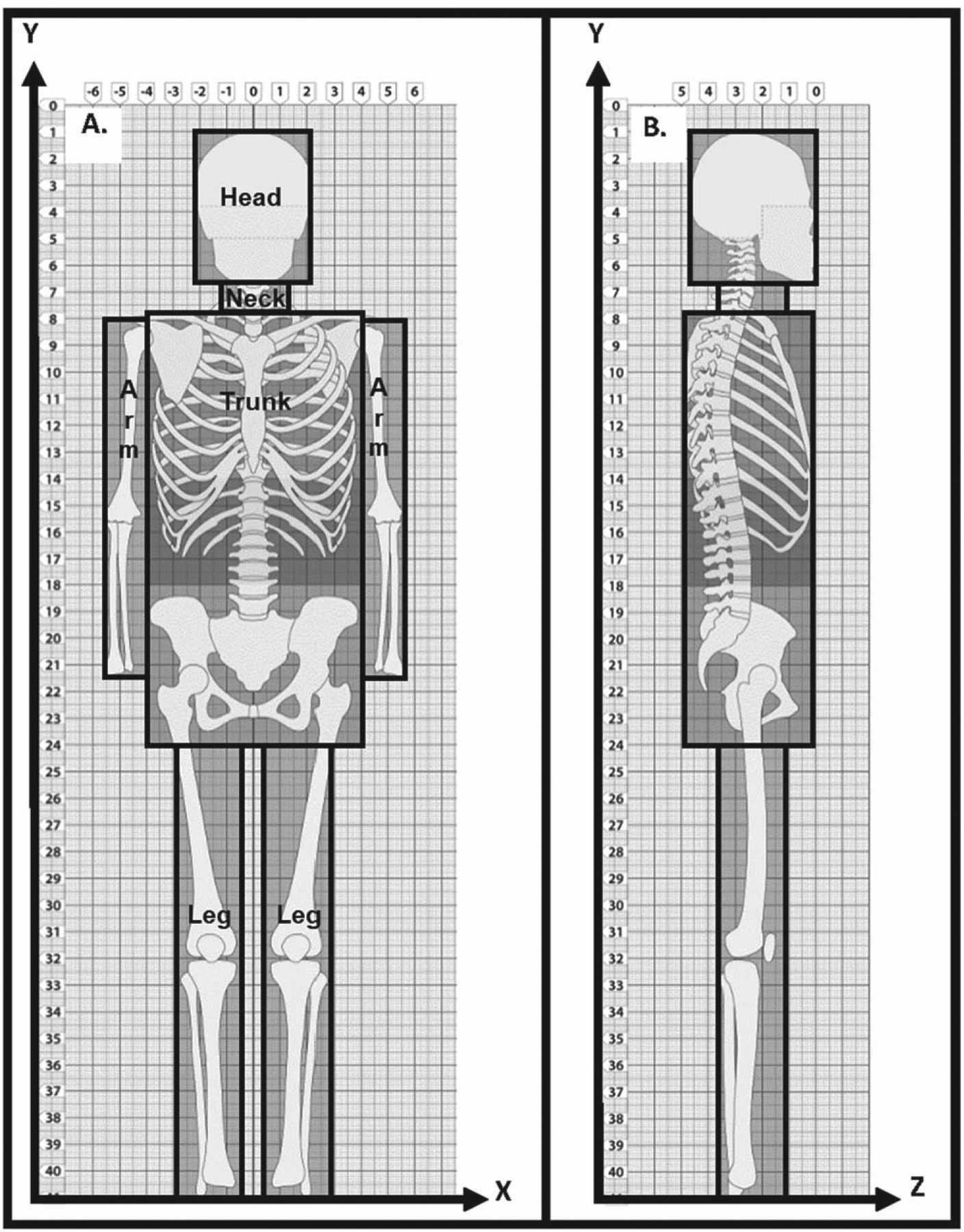
Diagrams of our computational phantom fitted to a 3D grid of points: (a) coronal view showing +x and −y axes and (b) sagittal view showing −y and −z axes. A skeleton is overlaid on the phantom for anatomic reference. The scalable body regions (head, neck, trunk, and extremities) are delineated in frontal view.

The MD Anderson Late Effects Group phantom was previously described by Howell *et al* (2019) and Stovall *et al*(2006). The phantom was built by bounding the body regions, i.e., head, neck, trunk, arms, and legs, of a generic gender-neutral adult skeleton (age = 18 years) by cuboids, which were then fit to a 3D grid of evenly spaced points (figure 1). Each cuboid is defined by its eight corner points obtained from its fit to the 3D grid. Various organs are defined within the phantom’s body regions as grids of points (Howell *et al* 2019).

### 2.2. Scaling functions

**Figure 2.**
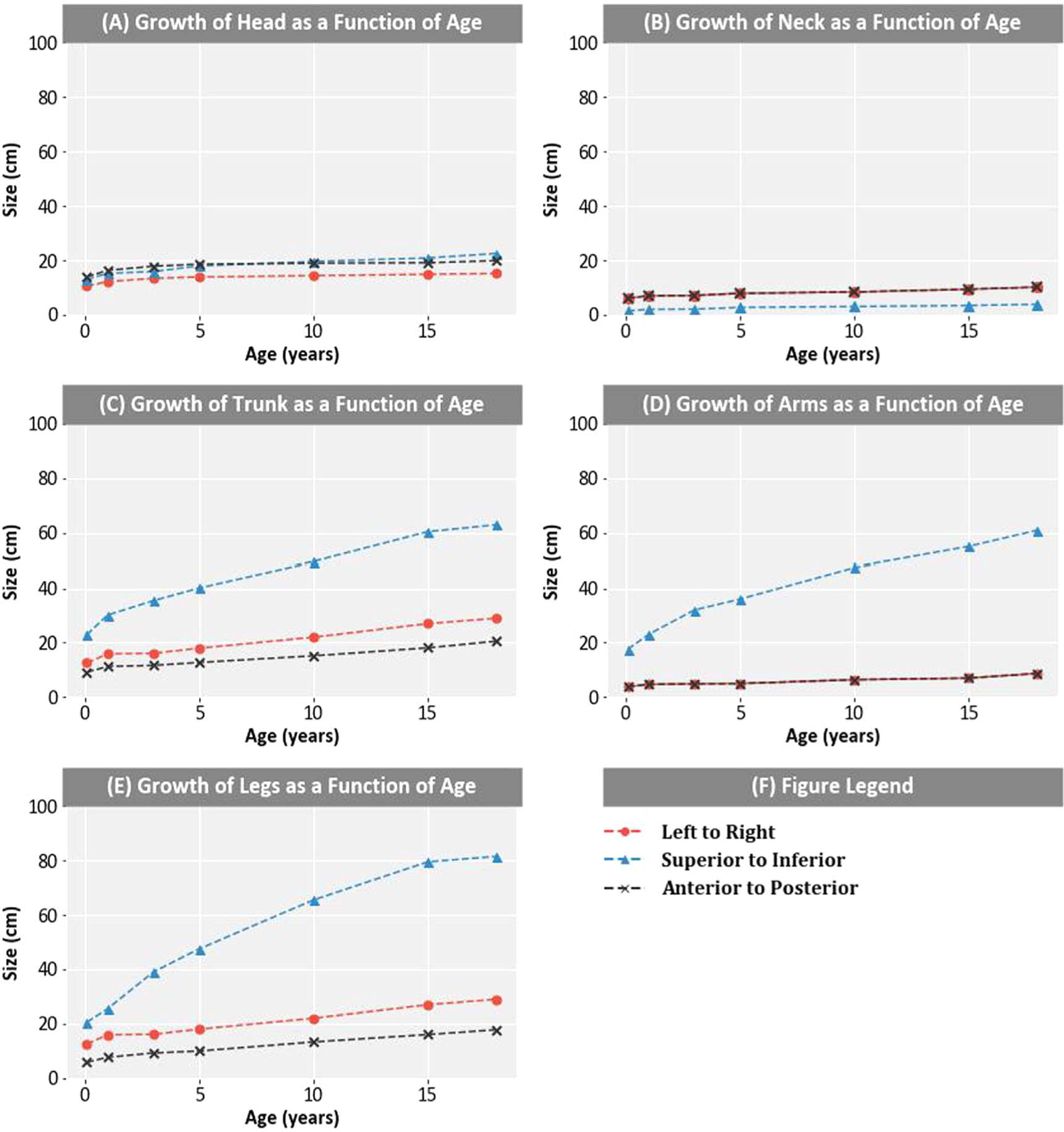
(A)–(E) Growth as a function of age from superior to inferior, left to right, and anterior to posterior for the head, neck, trunk, arms and legs for ages 1 month, 1, 3, 5, 10, 15, and 18 (adult) years (Snyder *et al* 1977, Huelke 1998).

Because this phantom was intended for use in late effects studies of cancer survivors, including children, whose ages at the time of their RT ranged from infant to adult, it was necessary to define scaling functions to adapt the generic adult phantom (18 years of age) to any age. The head, neck, trunk, and extremities of the human body undergo non-uniform growth from infant to adulthood. For example, at birth the human head makes up approximately one quarter of the total height, but that proportion decreases to about one-seventh by adulthood (Huelke 1998). This non-uniform growth was quantitatively reported by the Society of Automotive Engineers based on measurements of 4127 US infants, children and youths through 18 years old (Snyder *et al* 1977). As a function of age, using these growth data, we plotted sizes of the head, neck, trunk, and extremities (legs and arms) in three dimensions (figure 2). Then, we calculated the discrete scaling factor, F_dis_, by taking the ratio of the size of a specified body region r in a specified direction d at a given age a to its size in the generic phantom, equation (1).

(1) $${\text{F}}_{\text{dis}}(\text{d},\;\text{r},\;\text{a})\;=\;\frac{\text{S}(\text{d},\;\text{r},\;\text{a})}{\text{S}(\text{d},\;\text{r},\;g)},$$

where:

d ∈ {Left to right (x), superior to inferior (y), anterior to posterior (z)}

r ∈ {upper head (uh), lower head (lh), neck (n), trunk (tr), arms (ar), legs (lg)}

a ∈ {0.1 (1 month), 1, 3, 5, 10, 15, 18} and

g is a constant and is defined as age 18 years; the age of the generic phantom

Since equation (1) can only be used for scaling to discrete ages of 0.1 (1 month), 1, 3, 5, 10, 15, and 18 years, to allow scaling between these ages, we created age intervals of [0, 1), [1, 3), [3, 5), [5, 10), [10, 15), and [15, 18) and defined a continuous scaling function, for each age interval (equation (2)).

(2) $$\begin{array}{l} {\text{F}}_{\text{cont }}(\text{d},\;\text{r},\;\text{a})\;=\;{\text{F}}_{\text{dis}}\left(\text{d},\;\text{r},\;{\text{a}}_{-}\right)+\frac{\text{a}-{\text{a}}_{-}}{{\text{a}}_{+}-{\text{a}}_{-}}\\ \;\times \left({\text{F}}_{\text{dis}}\left(\text{d},\;\text{r},\;{\text{a}}_{+}\right)-{\text{F}}_{\text{dis}}\left(\text{d},\;\text{r},\;{\text{a}}_{-}\right)\right), \end{array}$$

Where a_−_ and a_+_ are lower and upper age bounds, respectively. Each organ was considered to follow the same scaling as the body region in which it was located.

### 2.3. Transformation functions

Each body region is represented by eight corner points and each organ is represented by a grid of points, where each point (P) is a set of three real numbers (coordinates) denoted by the variables x, y, and z, which represent the coordinates of a point. Once the necessary scaling factors are obtained using the scaling functions from section 2.2, we can apply these factors to each body region corner point and each organ point to transform them to various ages. Additionally, in the y and z directions, translations are also necessary so that all body regions remain contiguous and do not overlap as they scale; they are applied as described in the following paragraphs.

Since the generic phantom is symmetric about the *x*-axis (figure 1), the transformed x-coordinate (x_t_) can be obtained by taking the product of the continuous scaling factor (F_cont_) and the x-coordinate (x), equation (3).

(3) $$x_{\text{t}}\;=\;\text{X}\;\cdot \;{\text{F}}_{\text{cont}}(\text{x},\;\text{r},\;\text{a})$$

Conversely, the generic phantom is asymmetric about the *y*-axis and starts at the x-z plane where y = 1 cm (figure 1). To obtain the transformed y-coordinate (y_t_), we sum the product of the continuous scaling factor and the length for each body region (l_r,y_) along the *y*-axis of the generic phantom, equation (4). The length of each body region along the *y*-axis is obtained by taking the difference between the inferior (ibr) and superior (sbr) boundaries of that body region in the generic phantom, i.e. ${\text{y}}_{{\text{ibr}}_{\text{r}}}$ and ${\text{y}}_{{\text{sbr}}_{\text{r}}}$ respectively. For the body region in which the point lies, the length is the difference between the y-coordinate (y) and the superior boundary of that body region.

(4) $$y_{\text{t}}\;=\;\overset{\text{r}}{\underset{\text{r}=\text{uh}}{\sum }}1_{\text{r},\text{y}}\;\cdot \;{\text{F}}_{\text{cont}}(\text{y},\;\text{r},\;\text{a}),\;\text{where}\;1_{\text{r},\text{y}}\;=\;\{\begin{array}{cc} \text{y}\;-\;{\text{y}}_{{\text{sbr}}_{\text{r}},} & \text{y}\;\in \;\text{r}\\ {\text{y}}_{{\text{ibr}}_{\text{r}}}\;-\;{\text{y}}_{{\text{sbr}}_{\text{r}}}, & \text{y}\;\notin \;\text{r} \end{array}$$

Similarly, the generic phantom is asymmetric about the *z*-axis and the anterior aspect of each body region is at a different location in the x-y plane. Thus, to calculate a transformed z-coordinate (z_t_), first we calculate the difference between the z-coordinate (z) and the anterior boundary of the body region in the z direction $\left({\text{Z}}_{{\text{abr}}_{\text{r}}}\right)$. Next, we multiply this difference by the continuous scaling factor. Lastly, we add a shift (z_shift_), equation (5).

(5) $$z_{\text{t}}\;=\;\left(\text{Z}\;-\;{\text{Z}}_{{\text{abr}}_{\text{r}}}\right)\;\cdot \;{\text{F}}_{\text{cont}}(\text{Z},\text{r},\text{a})\;+\;{\text{z}}_{\text{shift}},$$

where z_shift_ is described in equation (6) and differs according to the body region,

(6) $${\text{z}}_{\text{shift}}\;=\;\frac{{\text{l}}_{\text{r}=\text{head},\text{z}}\;\cdot \;{\text{F}}_{\text{cont}}(\text{z},\text{uh},\;\text{a})\;-\;{\text{l}}_{\text{r},\text{z}}\;\cdot \;{\text{F}}_{\text{cont}}(\text{z},\;\text{r},\;\text{a})}{2},$$

where l_r,z_ is the length of the body region in the z direction.

### 2.4. Phantom adaptation from FORTRAN to DICOM

**Figure 3.**
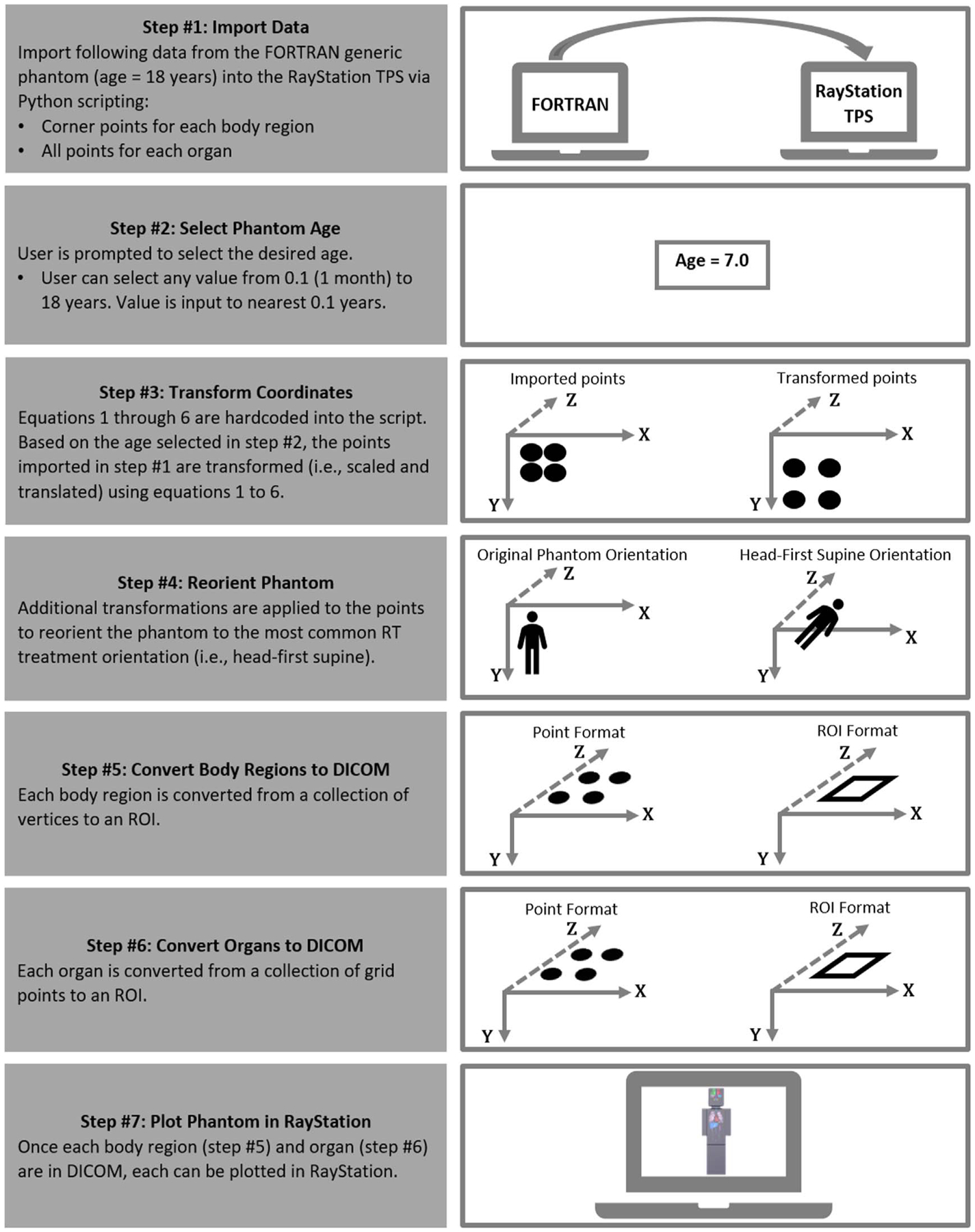
Flow chart explaining the adaptation of the phantom to DICOM standard.

For this study, we used RayStation V8.99 TPS (RaySearch Laboratories, Stockholm) because this TPS is used in our clinic and allows addition of user-specific customized features via Python scripting. We developed a script in Python that converts the generic phantom from FORTRAN to DICOM. The conversion is a 7-step process (figure 3), which is executed through a graphical user interface (GUI) scripted within the RayStation TPS.

1. Import Data: The corner points of each body region and the points of 9 different organs for the generic phantom (age = 18 years) are imported from the FORTRAN code into RayStation. The organs that were modeled in this study are—Brain (Frontal Lobes (Right and Left), Temporal Lobe (Right and Left), Parietal Lobe (Right and Left), Cerebellum, Occipital Lobes, and Inner brain), Heart, Liver, Lungs (Right and Left), Stomach, Pancreas, Kidneys (Right and Left), and Thyroid Lobes (Right and Left).
2. Select Phantom Age: The user is then prompted to select the desired age. The user can select any value from 0.1 (i.e., newborn) to 18 years. Values are specified to the nearest tenth of a year. We assume growth stops at age 18 and for ages above 18, phantom is simply scaled to age 18.
3. Transform Coordinates: Equations (1) through (6) are hardcoded into the script. Based on the phantom age selected in step 2, each point imported from step 1 is transformed using equations (1) through (6). After this step, each organ and body region will have been scaled to the appropriate size based on the user-selected age.
4. Reorient Phantom: Since the coordinate systems are defined differently in the FORTRAN code and RayStation TPS, we apply an additional rigid transformation to reorient the phantom to the most common RT treatment orientation, which is head-first supine.
5. Convert Body Regions to DICOM Standard: Each body region is converted from a collection of vertices to a region of interest (ROI) using RayStation’s Box ROI generation tool (through python script).
6. Convert Organs to DICOM Standard: Each organ is converted from a collection of grid points to an ROI using convex hull algorithm through python script.
7. Plot Phantom in RayStation: Once each body region and organ are in DICOM standard, each can be plotted in RayStation.

Once the phantom has been generated in RayStation, users can export the phantom in DICOM standard. This file can be uploaded into any DICOM-compatible TPS. A sample calculation of transformation of our generic ‘adult’ phantom to a 7-year-old phantom is illustrated in the appendix. Note that when we adapted our phantom from the FORTRAN to DICOM, we made two simplifications to the extremities: (1) the legs were simplified to consist of only one cuboid volume, as opposed to two separate cuboids and (2) the arm positions were constrained to a single position parallel to the sagittal plane (i.e. superior to inferior) as opposed to having variable positions of parallel or perpendicular to the sagittal plane of the body.

### 2.5. Validation

For this study, we performed two different validation approaches. For the first approach, we validated the conversion of our phantom model from FORTRAN to DICOM. To do this, we compared several geometric parameters between the phantom scaled to ages 1 month, 6 months, 1, 2, 3, 5, 8, 10, 15, and 18 years modeled in FORTRAN and DICOM. For the second approach, we compared the heights of the DICOM model of our phantom with population height data from the World Health Organization (WHO) and the Centers for Disease Control and Prevention (CDC) (Centers for Disease Control and Prevention 2000).

#### 2.5.1. Comparison of FORTRAN phantom with DICOM phantom

The first metric we calculated was the percent difference. This metric was calculated for all corner points in each spatial dimension of each body region and for the volumes of each body region between the phantom modeled in FORTRAN and DICOM. For the locations of the corner points, we calculated this difference in the x-, y-, and z-coordinates individually for each age-scaled phantom. This analysis was done for the head, neck, and trunk body regions, which includes the majority of organs of interest for late effects studies. Percent differences (PD) were calculated as follows:

(7) $$\text{PD}=\frac{\text{F}\;-\;\text{D}}{\text{F}}\;\times \;100\%,$$

where F corresponds to the coordinates (or volume) from FORTRAN (ground truth) and D corresponds to the coordinates (or volume) from DICOM for the specified body region.

The second metric we calculated was the normalized mean square distance (NMSD). The NMSD was calculated between the organs (heart, liver, lungs, stomach, and brain) for both phantoms using the following equation.

(8) $$\text{NMSD}=\frac{\sqrt{\sum_{i}^{\text{N}}{\left(x_{\text{F}}\;-\;x_{\text{D}}\right)}^{2}+{\left(y_{\text{F}}\;-\;y_{\text{D}}\right)}^{2}+{\left(z_{\text{F}}\;-\;z_{\text{D}}\right)}^{2}}}{\text{N}},$$

where, x, y, and z represent the coordinates of each organ point in the phantoms. The subscripts F and D represent that the coordinate of the point is from the FORTRAN and DICOM phantoms, respectively. N is the total number of points in each organ.

The third metric we calculated was the difference in heights between the FORTRAN and DICOM phantoms. This was calculated to ensure that the total height of the phantom was preserved when converted from FORTRAN to DICOM.

#### 2.5.2. Comparison of DICOM phantom with WHO/CDC population height data

In order to determine if the heights of our age-scaled phantoms were consistent with the heights of children across the ages of infant to adolescent, we compared our age-scaled phantom heights with a reference dataset. Specifically, we compared our age-scaled DICOM phantoms with the 50th-percentile heights reported by the WHO for ages 1 month, 6 months, and 1 year old and with the averages of the 50th-percentile heights for males and females reported by the CDC for ages 2, 3, 5, 8, 10, 15, and 18 years.

### 2.6. Comparison with reference phantoms organ masses

In FORTRAN format, organs in our phantom were modeled as grids of points, making it impossible to compare organ volumes or masses with other reference phantoms. In the updated DICOM format, such comparisons are possible and therefore were performed as part of this work. Specifically, we compared organ masses of the DICOM phantom with reference masses from from International Commission on Radiological Protection (ICRP) 89 (ICRP 2002) and University of Florida (UF)/National Cancer Institute (NCI) reference hybrid voxel phantoms for ages 6 days (newborn), 1, 5, 10, 15, and 18 years (Adult) (Lee *et al* 2010). We first calculated the organ masses for our DICOM phantoms (scaled to aforementioned ages) as a product of ICRU 46 reference densities and RayStation voxel based volumes. The volume of the organs in our DICOM phantom is independent of sex but the ICRP 89 and UF/NCI reference phantoms provide sex dependent masses for the 15 years old and adult phantoms. In those cases, the average of male and female reference organ masses were calculated. Additionally, for the heart and stomach, the UF/NCI reference phantoms have masses for the wall and contents of these organs. For kidneys, the masses of the medulla, pelvis and cortex were reported individually. For heart, stomach and kidneys, we calculated the total mass by summing the mass of each organ’s parts. Lastly, we computed the difference between the organ masses of the DICOM phantom and of the reference phantoms.

### 2.7. Dose calculation with DICOM phantom—Wilms’ tumor example

**Figure 4.**
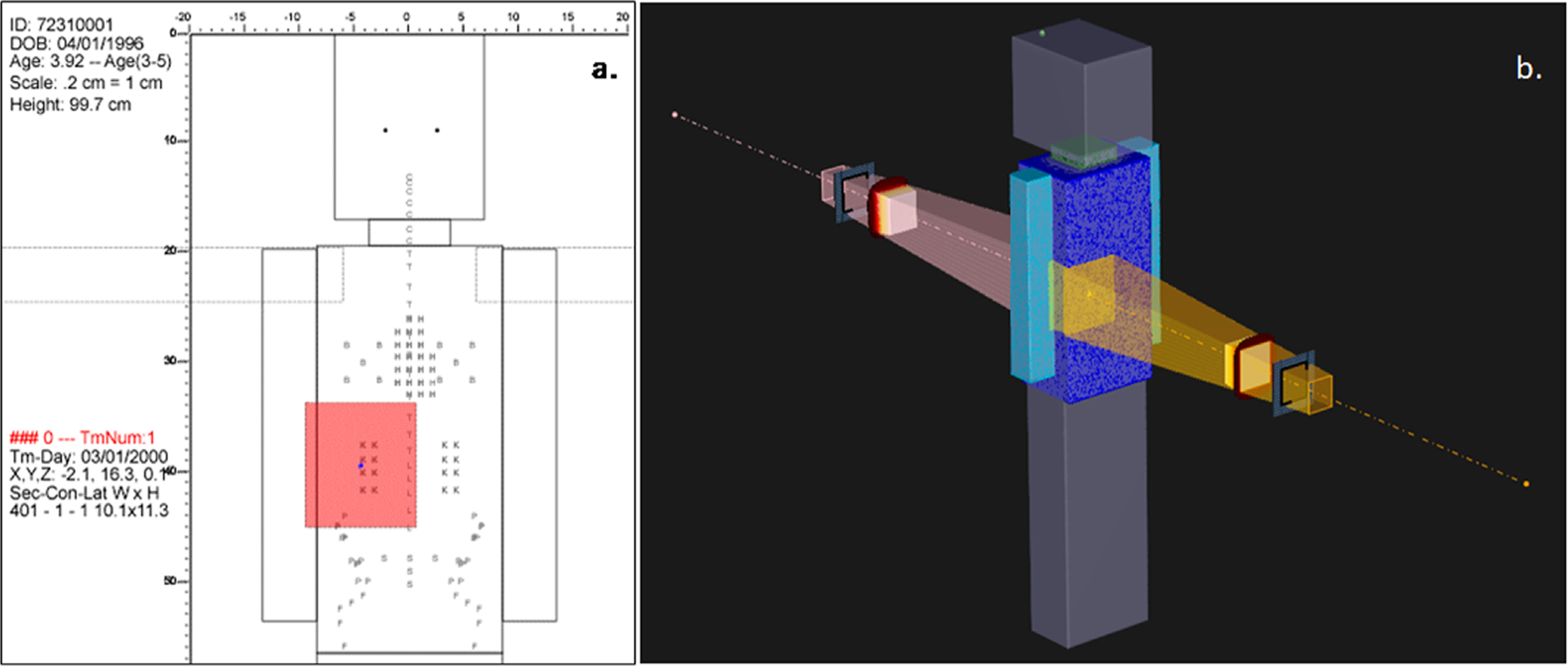
Right-sided AP/PA treatment fields simulated for Wilms’ tumor on a phantom scaled to 3.9 years in (a) FORTRAN and (b) DICOM formats. The coordinates of the field isocenters and field borders were the same in both planning systems.

To illustrate that our phantom can be used for dose calculations within a commercial TPS, we designed a treatment plan in RayStation and calculated dose to two organs at risk for our DICOM format phantom. The treatment plan was designed to be representative of a typical RT plan for an individual in the Childhood Cancer Survivor Study (CCSS) cohort. To do this, we selected a common type of paediatric cancer, Wilms’ tumor. We then performed a query of 7451 individuals in the CCSS expanded cohort (Leisenring *et al* 2009, Robison *et al* 2009) who received RT between 1985 and 1999 (data collected under IRB approved protocol and RT records previously abstracted). We identified 318 individuals diagnosed with Wilms’ tumor who were treated with anterior-to-posterior and posterior-to-anterior (AP/PA) directed abdominal flank fields. From these 318 treatments, we selected the median treatment field parameters to simulate a typical (6 MV) right-sided flank field RT plan: [1] age at RT: 3.9 years (range 0.45–20.9 years), target dose 10.80 Gy (range 1.08–36.72 Gy), [3] superior field border: diaphragm (N = 203), [4] Inferior border: L5 (N = 139). The right-sided AP/PA treatment fields are illustrated for a 3.9 year old phantom in FORTRAN and DICOM formats in figure 4. Doses to two organs at risk—the liver and pancreas—were calculated. Specifically, we calculated the mean dose received by each organ and the percentage of each organ that received dose ⩾5 Gy (V_5_). For comparison, we simulated the same treatment for our FORTRAN format phantom and calculated liver and pancreas doses using our in-house calculation method.

## 3. Results

### 3.1. Phantom modeled in DICOM standard

**Figure 5.**
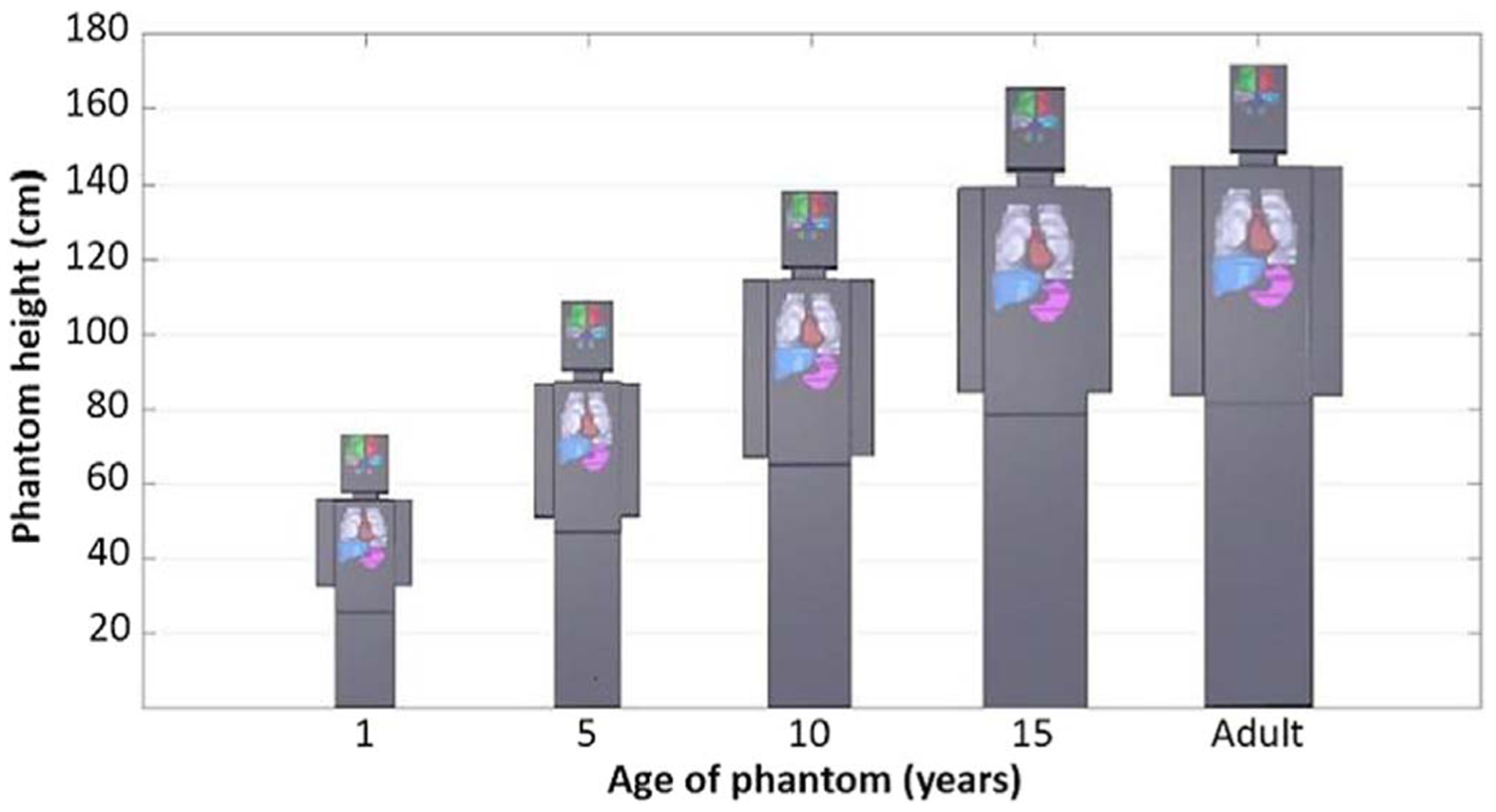
Illustration of TPS generated 3D renderings of age-scaled phantoms modeled in DICOM. Selected organs (brain, lungs, heart, liver, and stomach) were also rendered for each scaled phantom.

The age-scalable computational phantom modeled in DICOM standard is illistrated in figure 5, which includes 3D renderings of our phantom generated in RayStation TPS and scaled to ages 1, 5, 10, 15, and 18 (adult).

### 3.2. Comparison between phantom modeled in FORTRAN and DICOM

**Figure 6.**
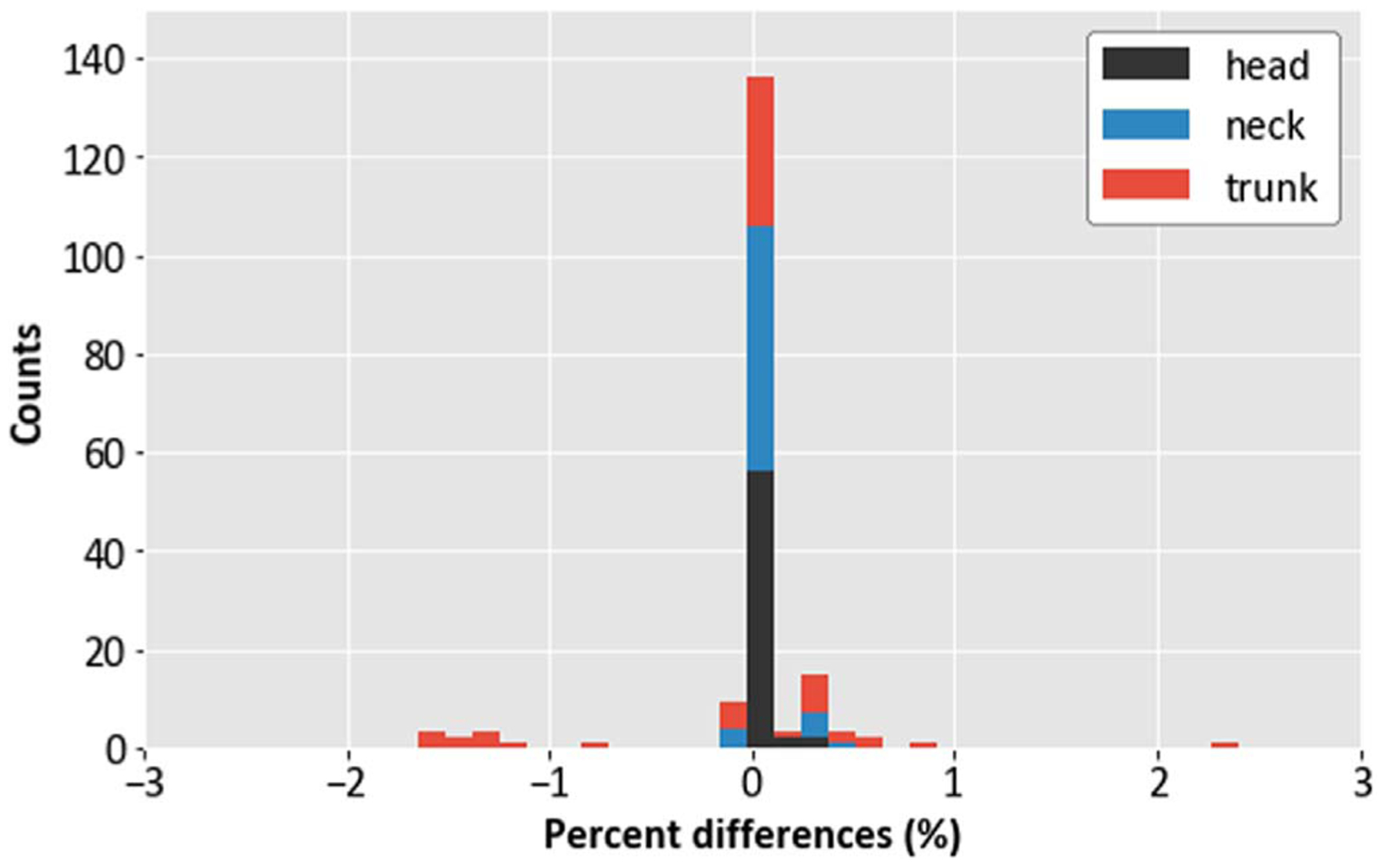
Histogram showing the frequency of percent differences in the corner points of body regions (excluding legs and arms) of the DICOM phantoms.

A histogram illustrating distribution and range of error in reproducing correct locations of body-region corner points is shown in figure 6. All observed differences were within 3%, with 0% being most frequently observed. The results of the percent difference calculations in the volumes of the head, neck, and trunk of the two phantoms are shown in table 1. For the volume analysis, we observed all differences within 3%.

The normalized mean square distance calulations resulted in strong agreement in the location of organs across the studied age range. The maximum NMSD was 7.80 × 10^−2^ mm for occipital lobe of age 1 month. When we compared the percent differences in the phantoms’ heights modeled in DICOM and FORTRAN, we found accurate agreement (difference = 0%) between the phantoms for ages 1, 3, 5, 10, and 15 years and a difference of 0.03% between the phantoms for age 18 years.

### 3.3. Comparison of DICOM phantom with population height data

**Figure 7.**
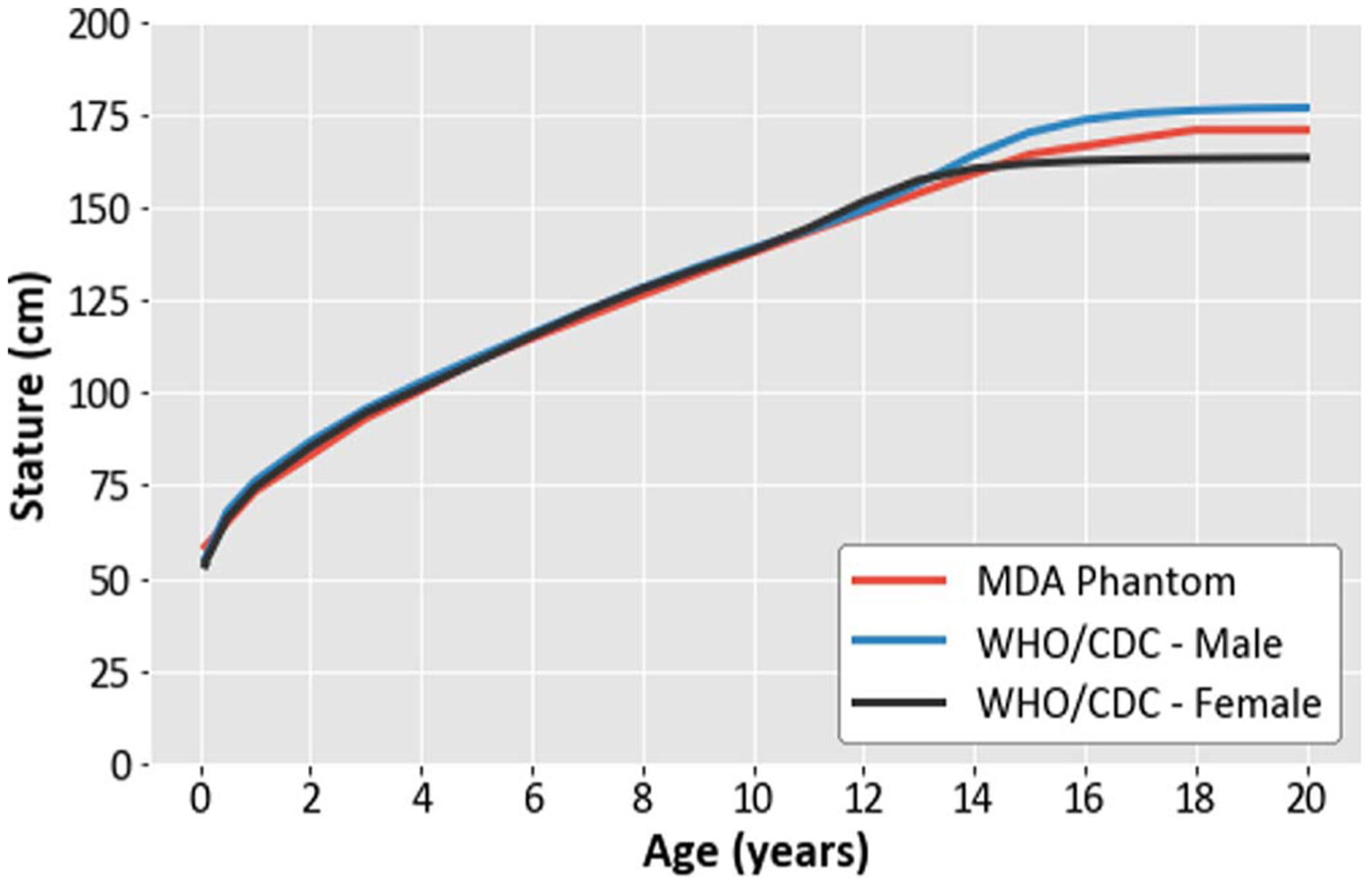
Comparison of the heights of the computational phantom modeled in DICOM with the WHO/CDC heights (Centers for Disease Control and Prevention 2000).

Figure 7 shows, for ages 1 month through 20 years, a comparison of the heights of the age-scaled DICOM phantoms with the averages of the 50th-percentile CDC reported heights for males and females. The differences were 3.6, 2.1, 0.3, 1.4, 0.6, 1.0, and 0.7% for ages 2, 3, 5, 8, 10, 15, and 18 years, respectively. For 1 month, 6 months, and 1 year, the differences between the DICOM phantom heights and the WHO 50th-percentile heights were 6.9, 3.1, and 2.6%, respectively.

### 3.4. Comparison of organ masses of DICOM phantom with ICRP 89, and UF/NCI reference hybrid phantom data

The masses of nine organs from our DICOM phantom are listed in table 2. Also reported in table 2, are the absolute differences between organ masses in our phantom and those reported for ICRP 89 and UF/NCI phantoms. The differences are all negative (apart from newborn brain), i.e., the organ masses in both reference datasets are substantially greater than the organ masses in our phantom.

### 3.5. Results from dose calculation (Wilms’ tumor example)

The V_5_ and mean dose (Gy) for liver and pancreas calculated with our in-house calculation system (with FORTRAN phantom) and the RayStation TPS (with DICOM phantom) are reported in table 3; percent difference in each case is also reported. The percent differences between mean doses for liver and pancreas were −4% and 1%, respectively. The percent differences between V_5_ values for liver and pancreas were −6% and 7%, respectively.

## 4. Discussion

In this study, we successfully adapted our phantom model from FORTRAN to DICOM, allowing for importation into any commercial TPS (RayStation, Eclipse, Pinnacle, Monaco, etc), where it can be used for a variety of dosimetry studies. Analogous to our FORTRAN phantom, our DICOM phantom can be scaled to any age and can be used to perform retrospective dose reconstructions for survivors treated with contemporary RT. In such studies doses to distant organs will need to be retrospectively reconstructed on computational phantoms because CT images used for treatment planning may not be available for epidemiologic studies, and even if they are, the CT data will be limited to anatomy near the target volume and will not include distant structures that are typically of interest in such epidemiologic studies. For example, for female paediatric brain cancer survivors, whose CT scans only included the head and possibly the neck regions, organs of interest for late effects studies may include the heart, breasts, and ovaries, for which anatomical information is not present in the CT scan. In such cases, our phantom can be scaled to any age at RT and co-registered with the patient CT scan, and then doses to other organs can be reconstructed using the methodologies described in previous studies (Stovall *et al* 2006, Howell *et al* 2019). An important reason for using our phantom in late effect studies for cohorts treated with contemporary RT is to facilitate comparison with cohorts treated with historic RT. The dosimetry for RT-related late effects studies in the literature has been predominantly conducted using the MD Anderson Late Effects Group phantom. Howell *et al* (2019) reports more than one hundred late effects studies for which the MD Anderson Late Effects Group performed dose reconstructions for cohorts with thousands of childhood cancer survivor studies, e.g. the CCSS, St. Jude Lifetime (Hudson *et al* 2011, Hudson *et al* 2017), Adult Life after Childhood Cancer in Scandinavia (Asdahl *et al* 2015), and Dutch Childhood Oncology Group (Teepen *et al* 2017). Furthermore, other reference phantoms, e.g., UF/NCI, while anatomically more realistic compared to our phantom, are only available for discrete integer ages and cannot be scaled to any arbitrary age.

Our validation studies showed that our phantom was correctly adapted from FORTRAN to DICOM. The histogram analysis of the percent differences between the corner points of head, neck, and trunk body regions and volumes of the body regions were in good agreement (within 3%) and the majority (94.4%) of corner points agreed within 1%. The DICOM model of the phantom consists of organ contours developed from a grid of points that were obtained after transforming the points of the FORTRAN model of the phantom. The points defining the organs were conserved quantitatively between the FORTRAN and DICOM models, with mean differences being less than 0.1 mm for all organ points. The maximum NMSD obtained was 7.80 × 10^−2^ mm for occipital lobe of age 1 month. The heights of our age-scaled phantom agreed with WHO/CDC data within 7% from infant to adult, with best agreement for ages 5 years and older (<2%).

By modelling our phantom in DICOM, and, in particular, by converting our phantom’s organs from grids of points to contours from which volume (and mass) could be derived, we were, for the first time, able to compare our phantom’s organs masses with those from other reference phantoms. The results of this analysis demonstrated that the organ masses of our DICOM phantom are much less than those in both reference phantoms. The differences were similar in magnitude for comparisons with ICRP 89 and UF/NCI reference phantoms because the UF/NCI phantoms were adjusted to match ICRP 89 data (Lee *et al* 2010). It was not unexpected that our organ masses would differ from more recently developed ICRP 89 and UF/NCI phantoms because the organs in our FORTRAN phantom, which were the basis of the organs in the DICOM phantom, were developed from crude sampling of organ points from cross-sectional anatomical images (Howell *et al* 2019). While the differences in mass were large, it is important to underscore that dosimetry conducted with our in-house dose calculation methodology and FORTRAN phantom did not use organ masses for calculations. In that system, we calculated doses to the individual points comprising an organ. Then from those data, the mean organ doses were taken as the mathematical average of the point doses. Similarly, dose-volume metrics were approximated from percentage of points, e.g., the V5 was estimated from the percentage of points with dose ⩾5 Gy.

In this study, we also illustrated that our phantom can be used for dose calculations within a commercial TPS. This example calculation demonstrated that our DICOM phantom can be scaled to any age (here 3.9 years), not just the ages illustrated in figure 1. Also, by selecting an example case that was typical of the types of calculations for which our in-house calculation method has been used, we were able to perform the same calculation for both the FORTRAN and DICOM format phantoms for direct comparison. Notably, we observed reasonably good agreement (within 7%) between the two calculation methods with the FORTRAN and DICOM phantoms (table 3). We attribute the differences between doses to the more accurate collapsed cone dose calculation algorithm in the RayStation TPS compared to the very simple 2D method used in our in-house calculation system.

While the DICOM model of our phantom was validated and can be used for dose calculations in a commercial TPS, the comparison of organ masses for our DICOM phantom and the organ masses from the reference phantoms revealed that our organs are too small and highlighted that refinement is necessary. The enhancement that we accomplished in this study, converting our phantom from FORTRAN to DICOM format, opens new avenues to achieve this. Namely, we can now register the DICOM model of our phantom with patients’ and other phantoms’ (Lee *et al* 2010) CT images to evaluate the correspondence of organs.

Phantoms enhancements that we are working towards include, redefining organs to be more anatomically realistic in size and shape and adding substructures to more organs. For example, the heart, an important organ for RT-related late cardiac disease, was developed using an anatomy atlas and was modeled as a 55 point grid with no substructures. We can enhance the shape and size of the heart based on the realistic anatomy and compare the new model with models from UF/NCI reference phantoms. The heart model in our phantom could be further refined by adding substructures and increasing the resolution of points that would enable calculations of RT doses to specific substructures. These substructure doses could be used to further enhance dose-response models for RT-related late cardiac disease, which at present are based on whole-heart doses (Mulrooney *et al* 2009, Haddy *et al* 2016, Bates *et al* 2019). Additionally, other organs in the FORTRAN phantom were designed with low resolution, e.g., kidneys and pituitary glands have 15 and 1 points, respectively. For these low-resolution organs, we were not able to create contoured volumes that can structurally represent the organ in the RayStation TPS. Finally, we are presently working on adding a colorectal model to our phantom to understand the relationship between dose to the colon/rectum (and its substructures) and treatment-related colorectal second cancers in childhood cancer survivors. Existing studies on this topic have not included detailed colorectal dosimetry (Henderson *et al* 2012, Nottage *et al* 2012, Tukenova *et al* 2012).

## 5. Conclusion

We successfully adapted our age-scalable computational phantom from the FORTAN language to DICOM standard, which can be imported into any commercial TPS. The modelling of the phantom in DICOM allows visualization of organs and body regions in three dimensions, which was not done before. Most importantly, the phantom modeled in DICOM can be used for late effects studies of cohorts that include survivors treated with contemporary RT.

## Figures and Tables

**Table 1.** Percent differences between the volumes of FORTRAN and DICOM phantom body regions.

	Age (years)									
Body regions	0.1 (1 month)	0.5 (6 months)	1	2	3	5	8	10	15	18
Head	0.8	0.0	0.0	0.0	0.0	0.0	0.0	0.0	0.0	0.0
Neck	1.9	0.1	0.0	0.3	0.3	0.1	0.4	0.0	0.2	0.2
Trunk	2.8	1.2	1.3	1.2	1.3	1.3	1.3	1.3	1.2	1.2

**Table 2.** Mass (in gram) of DICOM phantom organs and comparison with masses from ICRP 89 and UF/NCI reference hybrid voxel phantom data. In each case, the difference between DICOM phantom organ mass and ICRP 89 or UF/NCI reference masses were calculated.

	Newborn			1 year			5 years			10 years			15 years			18 years (Adult)		
Organs	DICOM	DICOM —ICRP 89	DICOM —UF ref.	DICOM	DICOM —ICRP 89	DICOM —UF ref.	DICOM	DICOM —ICRP 89	DICOM —UF ref.	DICOM	DICOM —ICRP 89	DICOM —UF ref.	DICOM	DICOM —ICRP 89	DICOM —UF ref.	DICOM	DICOM —ICRP 89	DICOM —UF ref.
Heart	12.27	−33.73	−13.68	24.62	−73.38	−73.45	42.39	−177.61	−176.98	76.62	−293.38	−292.54	135.68	−464.32	−462.82	173.26	−556.74	−555.45
Brain	449.40	69.40	128.27	651.56	−298.44	−298.25	900.89	−344.11	−343.83	1003.63	−306.37	−305.84	1083.11	−276.89	−274.11	1153.46	−221.54	−215.17
Liver	54.32	−75.68	−75.36	108.86	−221.14	−220.61	181.62	−388.38	−383.20	318.69	−511.31	−510.14	572.21	−727.79	−726.37	738.61	−861.39	−858.70
Lungs	41.43	−18.57	−18.30	84.32	−65.68	−65.44	146.05	−153.95	−152.58	259.93	−240.07	−238.22	460.98	−364.02	−361.87	590.71	−484.29	−481.42
Stomach	25.27	−21.73	−7.05	48.26	−38.74	−38.41	75.79	−57.21	−56.59	133.35	−68.65	−67.44	225.17	−94.83	−94.01	287.68	−97.32	−97.14
Pancreas	3.13	−2.87	−2.86	5.03	−14.97	−14.90	7.28	−27.72	−27.67	11.56	−48.44	−48.37	20.56	−84.44	−84.19	26.31	−103.69	−103.26
Kidneys	2.76	−22.24	−23.48	5.05	−64.95	−68.31	9.21	−100.79	−105.85	16.22	−163.78	−172.78	27.62	−217.38	−229.23	34.17	−258.33	−272.23
Thyroid	0.26	−1.04	−1.03	0.44	−1.36	−1.35	0.60	−2.80	−2.80	0.55	−7.35	−7.35	0.61	−11.39	−11.31	0.62	−17.88	−17.82

**Table 3.** Percent difference between the V_5_ and mean dose of DICOM and FORTRAN phantom organs.

	V_5_ (%)			Mean Dose (Gy)		
Organ at Risk	DICOM	FORTRAN	% Difference	DICOM	FORTRAN	% Difference
Liver	91%	96%	−6%	9.55	9.99	−4%
Pancreas	72%	67%	7%	7.57	7.50	1%

## Appendix

We present a sample calculation following the steps in figure 3 to illustrate how our generic ‘adult’ phantom is transformed to a 7.0-year-old phantom. Due to the symmetric nature of a cube, if two corner points describing a diagonal of the cube are known, then we can calculate the remaining six corner points of the cube. Hence, we only present the transformation of two opposite corner points for each of the phantom’s cuboidal body regions. Additionally, we illustrate the transformation of one point in an organ within the trunk body region.

### Step 1: Import data

In this step, we import the organ points and corner points of the body region from the FORTRAN generic phantom into a DICOM file present in RayStation. To begin, p_r_ and q_r_ represent the two opposite corners of the cube. Here r *ϵ* {upper head (uh), lower head (lh), neck (n), trunk (tr), arms (ar), legs (lg)}. The body regions with their opposite corner points are presented in the table below.

To illustrate how organ points are transformed, we chose the point (−3.40, 14.30, 2.60) in the liver.

### Step 2: Select phantom age

Here, we select the age of the phantom as 7.0 years.

**Table A1.** Opposite corner points describing the main diagonal of each body region in the generic phantom. The corner points are scaled and translated using equations (1) through (6) to obtain the phantom of age a.

Head (upper and lower)	p_uh_ (−2.30, 1.00, 0.00) and q_lh_(2.30, 6.70, 4.80)
Neck	p_n_ (−1.20, 6.70, 1.10) and q_n_(1.20, 7.60, 3.50)
Trunk	p_tr_ (−4.00, 7.60, 0.10) and q_tr_(4.00, 24.00, 4.90)
Leg	p_lg_ (−2.80, 24.00, 1.10) and q_lg_(2.80, 43.20, 3.60)
Arm^a^	p_ar_ (4.00, 8.00, 1.60) and q_ar_(5.60, 21.60, 3.20)

a In table A1, we present the calculation for the right arm only due to symmetry in approach.

**Table A2.** Scaling factors corresponding to the ages of 5.0 and 10.0 years are shown as age = 7.0 *ϵ* [5.0, 10.0).

	Age = 5.0 (LR (x), SI (y), AP (z))^a^	Age = 10.0 (LR (x), SI (y), AP (z))^a^
Head	(3.022, 3.286, 3.872)	(3.130, 3.464, 3.957)
Neck	(3.292, 3.026, 3.292)	(3.500, 3.410, 3.500)
Trunk	(2.250, 2.442, 2.653)	(2.750, 3.018, 3.163)
Leg	(3.750, 2.474, 3.840)	(4.583, 3.411, 5.120)
Arm	(3.125, 2.647, 3.125)	(4.000, 3.493, 4.000)

a LR = Left to Right, SI = Superior to Inferior, AP = Anterior to Posterior directions.

**Table A3.** Scaling factors for each body region to scale the phantom from generic to age 7.0 years.

	(LR (x), SI (y), AP (z))^a^
Head	(3.065, 3.357, 3.906)
Neck	(3.375, 3.180, 3.375)
Trunk	(2.450, 2.672, 2.857)
Leg	(4.083, 2.849, 4.352)
Arm	(3.475, 2.985, 3.475)

a LR = Left to Right, SI = Superior to Inferior, AP = Anterior to Posterior directions.

### Step 3: Transform coordinates

Based on the user-specified age, we use equations (1) through (6) to transform the points of the generic phantom to a phantom of age 7.0 years. The process can be divided into two phases, (i) calculation of the scaling factor and (ii) scaling/translation of generic phantom points.

In phase (i), we identify the age group and access the preloaded scaling factor data for the lower and upper age bounds. Here, the age of 7.0 years falls within the category [5, 10). To calculate the scaling factor for 7.0 years, we use equation (A2), which requires the scaling factors for upper and lower age bounds (a_−_). The scaling factors corresponding to a_−_ = 5.0 and a_+_ = 10.0 for each body region are presented in table A2.

(A2) $${\text{F}}_{\text{cont}}(\text{x},\text{uh},7)={\text{F}}_{\text{dis}}(\text{x},\text{uh},5)+\frac{7.0\;-\;5.0}{10.0-5.0}\;\times \;\left({\text{F}}_{\text{dis}}(\text{x},\text{uh},10)\;-\;{\text{F}}_{\text{dis}}(\text{x},\text{uh},5)\right){\text{F}}_{\text{cont}}(\text{x},\;\text{uh},\;7)=3.022+\frac{7.0\;-\;5.0}{10.0\;-5.0}(3.130\;-\;3.022)=3.065$$

The above calculation is repeated in all three directions for each body region, and we finally obtain all scaling factors for age 7.0 years (table A3). For the organ points, the same calculations are repeated based on the body region in which the organ point lies.

In phase (ii), the body region corner points and the organ points are transformed using equations (3)–(6). The transformation of each body region is shown in the subsections below.

#### Transformation of the head corner points.

For p_uh_(−2.30, 1.00, 0.00):

$$x_{\text{t}}=\text{x}\;\cdot \;{\text{F}}_{\text{cont}}(\text{x},\;\text{r},\;\text{a})=-2.30\;\cdot \;3.065=-7.05\;\text{cm}$$

$$y_{\text{t}}=\overset{\text{uh}}{\underset{\text{r}=\text{uh}}{\sum }}1_{\text{uh}}\;\cdot \;{\text{F}}_{\text{cont}}(\text{y},\text{uh},7)\;=\text{(y}\;-\;{\text{y}}_{{\text{sbr}}_{\text{uh}}})\cdot {\text{F}}_{\text{cont}}(\text{y},\;\text{uh},\;7\;=(1.00-1.00)\;\cdot \;3.357=0.00\;\text{cm}$$

$${\text{z}}_{\text{shift}}=\frac{{\text{Z}}_{\text{head}}\cdot \text{F}(\text{z},\text{uh},7)-{\text{Z}}_{\text{head}}\cdot \text{F}(\text{z},\text{uh},7)}{2}\;=0.00$$

$$z_{\text{t}}=\left(\text{z}\;-\;{\text{z}}_{\text{ab}r_{\text{uh}}}\right)\;\cdot \;\text{F}(\text{z},\text{uh},7)+{\text{z}}_{\text{shift}}\;0.00\;\cdot \;3.906=0.00\;\text{cm}$$

For q_lh_(2.30, 6.70, 4.80):

$$x_{\text{t}}=2.30\;\cdot \;3.065=7.05\;\text{cm}$$

$$y_{\text{t}}=\left({\text{y}}_{{\text{ibr}}_{\text{uh}}}\;-\;{\text{y}}_{\text{sbruh}}\right)\;\cdot \;{\text{F}}_{\text{cont}}(\text{y},\text{uh},7)+\left(\text{y}\;-\;{\text{y}}_{{\text{ibr}}_{\text{uh}}}\right)\;\cdot \;{\text{F}}_{\text{cont}}(\text{y},\text{n},7)=(3.80\;-\;1.00)\;\cdot \;3.357+(6.70-3.80)\;\cdot \;3.180=18.62\;\text{cm}$$

$${\text{z}}_{\text{shift}}=\frac{{\text{Z}}_{\text{head}}\cdot \text{F}(\text{z},\text{uh},7)-{\text{Z}}_{\text{head}}\cdot \text{F}(\text{z},\text{uh},7)}{2}\;=0.00$$

$$z_{\text{t}}=4.80\;\cdot \;3.906=18.75\;\text{cm}$$

#### Transformation of the neck corner points.

For p_n_(−1.20, 6.70, 1.10):

$$x_{\text{t}}=-1.20\;\cdot \;3.375=-4.05\;\text{cm}$$

Since the superior boundary of the neck on the *y-*axis is same as the inferior boundary of the lower head, the transformed point is same as that obtained for the lower boundary of the head on the *y*-axis.

$${\text{z}}_{\text{shift}\;}=\frac{{\text{Z}}_{\text{head}\;}\cdot \text{F}(\text{z},\text{uh},\text{a})-Z_{\text{n}}\cdot \text{F}(\text{z},\text{n},\text{a})}{2}\;=\frac{(4.70-0.00)\cdot 3.906-(3.50-1.10)\cdot 3.375}{2}\;=5.13\;\text{cm}$$

$$z_{\text{t}}=(1.10-1.10)\cdot 3.375+5.13=5.13\;\;\text{cm}$$

For q_n_(1.20, 7.60, 3.50):

$$x_{\text{t}}=1.20\cdot 3.375=4.05\;\text{cm}$$

$$y_{\text{t}}=\left({\text{y}}_{{\text{ibr}}_{\text{uh}}}-{\text{y}}_{{\text{sbr}}_{\text{uh}}}\right)\cdot {\text{F}}_{\text{cont}}(\text{y},\text{uh},7)+\left({\text{y}}_{{\text{ibr}}_{\text{lh}}}-{\text{y}}_{{\text{sbr}}_{\text{lh}}}\right)\;\cdot {\text{F}}_{\text{cont}}(\text{y},\text{n},7)+\left(\text{y}-{\text{y}}_{{\text{sbr}}_{\text{n}}}\right)\cdot {\text{F}}_{\text{cont}}(\text{y},\text{n},7)\;=(3.80-1.00)\cdot 3.357+(6.70-3.80)\cdot 3.180\;+(7.60-6.70)\cdot 3.180=21.48\;\text{cm}$$

$$z_{\text{t}}=(3.50-1.10)\cdot 3.375+5.13=13.23\;\text{cm}$$

$$z_{\text{t}}=(1.60-1.60)+\frac{(4.70-0.00)\cdot 3.906-(3.20-1.60)\cdot 3.475}{2}\;=6.40\;\text{cm}$$

#### Transformation of the trunk corner points.

For p_tr_(−4.00, 7.60, 0.10):

$$x_{\text{t}}=-4.00\cdot 2.450=-9.80\;\text{cm}$$

$$y_{\text{t}}=21.48\;\text{cm}$$

$${\text{Z}}_{\text{shift}\;}=\frac{4.70\cdot 3.906-4.90\cdot 2.857}{2}=2.18\;\text{cm}$$

$$z_{\text{t}}=(0.10-0.10)\cdot 2.857+2.18=2.18\;\text{cm}$$

For q_tr_(4.00, 24.00, 4.90):

$$x_{\text{t}}=4.00\cdot 2.450=9.80\;\text{cm}$$

$$y_{\text{t}}=\left({\text{y}}_{{\text{ibr}}_{\text{uh}\;}}-{\text{y}}_{{\text{sbr}}_{\text{uh}}\;}\right)\cdot {\text{F}}_{\text{cont}\;}(\text{y},\text{uh},7)+\left({\text{y}}_{{\text{ibr}}_{\text{lh}\;}}-{\text{y}}_{{\text{sbr}}_{\text{lh}}\;}\right)\;\cdot {\text{F}}_{\text{cont}\;}(\text{y},\text{n},7)+\left({\text{y}}_{{\text{ibr}}_{\text{n}}}-{\text{y}}_{{\text{sbr}}_{\text{n}}}\right)\cdot {\text{F}}_{\text{cont}\;}(\text{y},\text{n},7)\;+\left(\text{y}-{\text{y}}_{{\text{sbr}}_{\text{t}}}\right)\cdot {\text{F}}_{\text{cont}\;}(\text{y},\text{tr},7)=(3.80-1.00)\;\cdot 3.357+(6.70-3.80)\cdot 3.180\;+(7.60-6.70)\cdot 3.180+(24.00-7.60)\;\cdot 2.672=65.30\;\text{cm}$$

$$z_{\text{t}}=(4.90-0.10)\cdot 2.857+2.18=15.89\;\text{cm}$$

#### Transformation of the leg corner points.

For p_lg_(−2.80, 24.00, 1.10) and q_lg_(2.80, 43.20, 3.60):

The transformed x- and z-coordinates of the leg volume are same as the transformed x- and z-coordinates of the trunk. This was done because we have one leg volume in the phantom. To prevent the leg volume lying outside the xz plane of the trunk when the phantom is scaled to higher ages, we used the same scaling in the xz direction as for the trunk. The length of the leg in the y direction is calculated using equation (4).

For p_lg_(−2.80, 24.00, 1.10):

$$y_{\text{t}}=65.30\;\text{cm}$$

For q_lg_(2.80, 43.20, 3.60):

$$y_{\text{t}}=\left({\text{y}}_{{\text{ibr}}_{\text{uh}\;}}-{\text{y}}_{{\text{sbr}}_{\text{uh}}\;}\right)\cdot {\text{F}}_{\text{cont}\;}(\text{y},\text{uh},7)+\left({\text{y}}_{{\text{ibr}}_{\text{Ih}\;}}-{\text{y}}_{{\text{sbr}}_{\text{Ih}}\;}\right)\;\cdot {\text{F}}_{\text{cont}\;}(\text{y},\text{n},7)+\left({\text{y}}_{{\text{ibr}}_{\text{n}}}-{\text{y}}_{{\text{sbr}}_{\text{n}}}\right)\cdot {\text{F}}_{\text{cont}\;}(\text{y},\text{n},7)\;+\left({\text{y}}_{{\text{ibr}}_{\text{tr}}}-{\text{y}}_{{\text{sbr}}_{\text{tr}}}\right)\cdot {\text{F}}_{\text{cont}\;}(\text{y},\text{tr},7)+\left(\text{y}-{\text{y}}_{{\text{sbr}}_{\text{lg}}}\right)\cdot \;{\text{F}}_{\text{cont}\;}(\text{y},\text{lg},7)=(3.80-1.00)\cdot 3.357\;+(6.70-3.80)\cdot 3.180+(7.60-6.70)\;\cdot 3.180+(24.00-7.60)\cdot 2.672\;+(43.20-24.00)\cdot 2.894=120.01\;\text{cm}$$

#### Transformation of the arm corner points.

For p_ar_(4.00, 8.00, 1.60):

On the *x*-axis, the arm starts at the same x-coordinate at which the trunk volume ends. Thus, x_t_ = 9.80 cm. On the *y*-axis, the volume starts at the same y-coordinate as the trunk.

For q_ar_(5.60, 21.60, 3.20):

$$x_{\text{t}}=5.60\cdot 3.475=19.46\;\text{cm}$$

$$y_{\text{t}}=\left({\text{y}}_{{\text{ibr}}_{\text{uh}}}-{\text{y}}_{{\text{sbr}}_{\text{sr}}}\right)\cdot {\text{F}}_{\text{cont}}(\text{y},\text{uh},7)+\left({\text{y}}_{{\text{ibr}}_{\text{rh}}}-{\text{y}}_{{\text{sbr}}_{\text{lh}}}\right)\;\cdot {\text{F}}_{\text{cont}\;}(\text{y},\text{n},7)+\left({\text{y}}_{{\text{ibr}}_{\text{n}}}-{\text{y}}_{{\text{sbr}}_{\text{n}}}\right)\cdot {\text{F}}_{\text{cont}\;}(\text{y},\text{n},7)\;+(\text{y}-{\text{y}}_{{\text{sbr}}_{\text{ar}})}\cdot {\text{F}}_{\text{cont}}(\text{y},\text{ar},7)=(3.80-1.00)\;\cdot 3.357+(6.70-3.80)\cdot 3.180\;+(7.60-6.70)\cdot 3.180+(21.60-8.00)\;\cdot 2.985=62.08\;\text{cm}$$

$$z_{\text{t}}=(3.20-1.60)\cdot 3.475+6.40=11.96\;\text{cm}$$

#### Transformation of organ points.

The scaling and translation of organs is performed in the same way as the body regions in which they are located. For example, we present here the scaling of one of the points of the liver, which is present in the trunk region. The sample calculation for the point (−3.40, 14.30, 2.60) in the liver is shown below:

$$x_{\text{t}}=-3.40\cdot 2.450=-8.33\;\text{cm}$$

$$y_{\text{t}}=(3.80-1.00)\cdot 3.357+(6.70-3.80)\cdot 3.180\;+(7.60-6.70)\cdot 3.180+(14.30-7.60)\;\cdot 2.672=39.39\;\text{cm}$$

$$z_{\text{t}}=(2.60-0.10)\cdot 2.857\;+\frac{4.70\cdot 3.906-4.90\cdot 2.857}{2}=9.32\;\text{cm}$$

Hence, the transformed point of the liver is (−8.33, 39.39, 9.32).

### Step 4: Reorient phantom

To model the phantom in the treatment planning system, we reorient our phantom to head-first, supine (HFS). Thus, the y- and z-coordinates are reversed. This results in the patient’s left to right orientation toward −x direction, superior to inferior orientation toward −z direction, and anterior to posterior orientation toward +y direction. An example of the calculated point of the liver in HFS orientation is presented below.

$$\left(-8.33,39.39,9.32)\;\;\underset{\to }{\text{y }\;\text{and}\;\text{z}\;\text{are}\;\text{flipped}}\;\;(-8.33,9.32,-39.39\right)$$

All organ points and body region corner points are reoriented in the same manner.

### Steps 5, 6, and 7: Convert body regions and organs to ROI/DICOM standard and plot phantom in RayStation

After the data are oriented in the HFS coordinate system, each body region is converted from a collection of points to ROI format. Likewise, the collection of points representing an organ is converted to ROI format. Next, the body regions and organs are plotted in RayStation. RayStation allows users to export the phantom in DICOM standard, which can be imported for use in any other TPS.
